# Examining the Effect of Supervisors’ Humble Leadership on Immediate and Delayed Well-Being in Postgraduate Students

**DOI:** 10.3390/bs14111004

**Published:** 2024-10-30

**Authors:** Zhihui Duan, Qi Zeng, Xin Liu

**Affiliations:** 1 School of Public Administration and Policy, Renmin University of China, Beijing 100872, China; duanzhihui@ruc.edu.cn; 2 College of Management, Zhongkai University of Agriculture and Engineering, Guangzhou 510225, China; zengqi@zhku.edu.cn

**Keywords:** supervisors’ humble leadership, postgraduate students’ well-being, basic psychological need satisfaction, power distance orientation

## Abstract

Despite supervisors playing a crucial role in the cultivation of postgraduate students, the impact of supervisors’ leadership on postgraduate students’ well-being is poorly understood. Based on Self-Determination Theory (SDT), this study explores the immediate and delayed effect of supervisors’ humble leadership on postgraduate students’ well-being, together with the mediating effects of basic psychological need satisfaction and the moderating effects of power distance orientation. Using a survey experiment (Study 1) and multi-timepoint questionnaire (Study 2) design, this paper finds that supervisors’ humble leadership influences postgraduate students’ well-being through its effect on basic psychological need satisfaction. Power distance orientation moderates the relationships between supervisors’ humble leadership, basic psychological need satisfaction, and postgraduate students’ well-being; specifically, humble leadership leads to higher basic psychological need satisfaction and well-being in students with high power distance orientation compared to those with low power distance orientation. This research validates the application of SDT in higher education and clarifies how supervisors’ humble leadership influences postgraduate students’ well-being, providing practical guidance for its improvement.

## 1. Introduction

As the scale of higher education in China continues to expand, postgraduate students face increasing pressures, resulting in various mental health issues [1]. Media reports frequently document psychological crises among Chinese postgraduate students [2], particularly, extreme cases of depression, anxiety, and suicide, often linked to inappropriate supervisor leadership, such as excessive criticism, lack of support, and overcontrol [3]. These incidents have drawn significant attention to the mental health of postgraduate students. However, existing research has predominantly focused on the prevention and intervention of mental health crises, with limited emphasis on the positive aspects of mental health [4,5]. In light of the development of positive psychology, Suldo and Shafferr [6] introduced the “dual-factor model of mental health”, which posits that complete mental health is not only the absence of emotional and behavioral problems, but also the presence of well-being. Moreover, studies have shown that well-being fosters a positive outlook, enhances creativity, and equips postgraduate students to better cope with academic pressures [7,8,9]. We feel it essential to examine postgraduate students’ mental health from a positive perspective and explore factors that contribute to their well-being.

Research has identified supervisors as key determinants of postgraduate students’ well-being [2,10,11], highlighting the crucial role of supervisor leadership in students’ development and mental and physical health [12]. However, the direct impact of supervisor leadership on postgraduate students’ well-being remains insufficiently explored. Existing studies have examined supervisor-student relationships and supportive behaviors, providing indirect evidence of leadership’s potential influence on student well-being. Supervisors who show respect, acknowledge achievements, provide constructive feedback [13], offer care and support [11], and cultivate harmonious supervisor-student relationships [10] significantly enhance postgraduate students’ well-being. From the perspective of leadership theories, these behaviors align with the characteristics of humble leadership [14].

In supervisor-centric research teams, postgraduate students, under the mentorship of their supervisors, assist in completing supervisor-led research projects while also working on their own dissertations [12]. During the research process, most students receive regular financial compensation from their supervisors [12]. Moreover, postgraduate students’ academic achievements and career success are significantly influenced by their supervisors [15]. From this perspective, the supervisor-student relationship can be understood as a distinctive hierarchical dynamic. Leadership theories, traditionally applied in organizational contexts, can be adapted to examine supervisory roles in academia [16]. Accordingly, this study draws on humble leadership theory to investigate how supervisors’ humble leadership influences postgraduate students’ well-being.

According to Self-Determination Theory (SDT), basic psychological need satisfaction serves as a key mediator linking the external environment (such as supervisors’ humble leadership) to individual well-being [17]. When the external environment facilitates basic psychological need satisfaction, well-being is enhanced, whereas its absence leads to a decline in well-being [17]. Teacher guidance plays a critical role in fostering basic psychological need satisfaction, thereby promoting well-being [18]. Therefore, it is reasonable to infer that supervisors’ humble leadership directly impacts basic psychological need satisfaction, which in turn influences postgraduate students’ well-being. Furthermore, SDT’s cross-cultural model suggests that the influence of external factors on basic psychological need satisfaction may vary according to cultural values [19]. Power distance orientation, as a key cultural value, may shape how postgraduate students respond to supervisory behaviors [20]. Consequently, power distance orientation could moderate the extent to which basic psychological need satisfaction is met under supervisors’ humble leadership and mediate the effect of supervisors’ humble leadership on postgraduate students’ well-being through basic psychological need satisfaction.

## 2. Theoretical Background and Hypotheses

### 2.1. Supervisors’ Humble Leadership and Postgraduate Students’ Well-Being

Humble leadership refers to supervisors who are grounded and adopt a ‘bottom-up’ approach to evaluating themselves and others [21]. The theory of humble leadership comprises three key behavioral dimensions: the demonstrated willingness to self-assess accurately, the recognition and appreciation of others’ strengths and contributions, and a commitment to continuous learning [14]. In contrast to traditional ‘top-down’ leadership, humble leadership prioritizes communication, mutual learning, and development between supervisors and subordinates [22]. Within the academic context, supervisors’ humble leadership involves objectively evaluating their own knowledge and limitations, appreciating students by affirming their strengths and contributions, and actively learning from students by embracing new ideas and suggestions. 

Well-being relates to experiences of psychological health and life satisfaction [17], representing both the ultimate aspiration of human society and a core objective of education [23]. For postgraduate students, well-being encompasses a holistic experience that reflects their ongoing personal and academic development [24]. This means that their well-being is shaped not only by positive subjective experiences derived from their academic and personal life, but also by a sense of psychological fulfillment through self-actualization. Following Deci and Ryan’s [17] definition, this study views postgraduate students’ well-being as more than just positive emotional states; it involves the organism’s capacity to experience vitality, psychological flexibility, and a profound inner sense of well-being.

According to SDT, an autonomously supportive environment—one in which superiors acknowledge subordinates’ perspectives, provide meaningful information without manipulation, offer choices, and encourage self-motivation [25]—is a crucial factor in promoting individual well-being [26]. Empirical studies suggest that humble leadership, as a form of autonomy support, creates a supportive environment that enhances individual well-being [27,28]. Specifically, individuals in such environments experience humble leadership through supervisor behaviors like openly acknowledging limitations in knowledge and skills [29], actively seeking feedback, and genuinely valuing others’ efforts and strengths [14]. This leadership style fosters trust and support from supervisors [30,31,32], contributing to the development of positive supervisor-subordinate relationships [21]. In academic settings, we suggest that these same principles apply, as supervisors who adopt humble leadership can create a supportive environment and build trustful supportive relationships with their students. Furthermore, the trust and support of supervisors, as well as positive supervisor-student relationships, have been proven to be important sources of postgraduate students’ well-being [2,11,33]. Based on this, we propose the following:

**Hypothesis** **1** **(H1).** *Supervisors’ humble leadership has a significant positive effect on postgraduate students’ well-being.*

### 2.2. The Mediating Role of Basic Psychological Need Satisfaction

Basic psychological needs are inherent in individuals and are essential nutrients for psychological development and the enhancement of well-being. These needs encompass autonomy, competence, and relatedness [17]. Autonomy refers to the individual’s need for self-directed action and independent choice; competence denotes the need to feel effective in achieving desired outcomes; and relatedness represents the need to form close and meaningful connections with others [19]. SDT emphasizes that basic psychological need satisfaction is a crucial mechanism linking the external environment with individual psychological states [17]. When the external environment meets these basic psychological needs, individuals exhibit positive psychological states, such as well-being.

SDT posits that basic psychological needs are not naturally satisfied, but require nurturance and support from the external environment [17]. A series of studies indicate a direct relationship between humble leadership and basic psychological need satisfaction [34,35]. In terms of autonomy needs, humble leadership values subordinates’ psychological freedom, allowing them to “be themselves” at work, thus enhancing their autonomy [36]. Regarding competence needs, humble leadership values individual contributions, appreciates their strengths, solicits their feedback, and acknowledges uncertainties, helping them discover their unique abilities [28]. In terms of relatedness needs, humble leadership is oriented towards relational identity [37], facilitating the development of close relationships between supervisors and subordinates [21]. Further, the satisfaction of these three types of basic psychological needs is holistic, where the fulfillment of one type of need facilitates the realization of other basic psychological needs [38].

Basic psychological need satisfaction is key to individual well-being [17]. Current research also reveals a significant link between basic psychological need satisfaction and well-being. Van den Broeck [39], through a meta-analysis, found significant correlations between basic psychological needs and well-being. Other empirical studies also show a strong and consistent correlation between basic psychological need satisfaction and well-being, which is capable of transcending a variety of cultural and economic backgrounds [40] and is consistently applicable across different research subjects [41].

In summary, existing research has elucidated the relationship between humble leadership and basic psychological need satisfaction, as well as the link between basic psychological need satisfaction and well-being. Although discussions on how supervisors’ humble leadership influences postgraduate students’ well-being through basic psychological need satisfaction are sparse, existing studies have found that effective teacher leadership facilitates basic psychological need satisfaction [18]. These studies also confirm that basic psychological need satisfaction partially or fully mediates the relationship between teacher autonomy support and individuals’ well-being [42]. Consequently, we posit the following:

**Hypothesis** **2** **(H2).** *Supervisors’ humble leadership influences postgraduate students’ well-being through its effect on basic psychological need satisfaction*.

### 2.3. The Moderating Role of Power Distance Orientation

SDT proposes that sociocultural factors modulate the effect of autonomy-supportive environments on psychological need satisfaction, thus shaping individual well-being [19]. Power distance, as a cultural value, reflects a society’s acceptance of power inequality [43]. At the individual level, power distance orientation represents individuals’ beliefs about status, authority, and power in organizational contexts, and reflects differences in their personal values and psychological traits [44]. Power distance orientation is considered a relatively stable characteristic that influences how individuals perceive and respond to supervisors’ behaviors [44]. Individuals with high power distance orientation tend to accept hierarchical relationships, acknowledge status differences, and are inclined to follow authority figures’ guidance. In contrast, individuals with low power distance orientation prefer equality and seek more interpersonal interaction with their leaders. 

Based on this, we predict that power distance orientation moderates the relationship between supervisors’ humble leadership and basic psychological need satisfaction. Individuals with high power distance orientation trust and respect experienced supervisors and actively interpret and accept their guidance [20]. When engaging with a humble supervisor, postgraduate students with high power distance orientation respond more positively, feel respected, and gain a stronger sense of competence, thereby enhancing their basic psychological need satisfaction. Furthermore, subordinates with high power distance orientation do not perceive respect and equal treatment from supervisors as given or expected; when receiving unexpected respect and equal treatment from a supervisor (such as supervisors’ humble leadership), their psychological reactions are more sensitive, and their basic psychological need satisfaction is higher [45].

In comparison, individuals with low power distance orientation are more concerned with equality in their relationship with supervisors [46]. Thus, supervisors’ humble leadership aligns with the cultural expectations of students with low power distance orientation, promoting the fulfillment of their basic psychological need satisfaction. However, this group tends to relatively overlook changes in supervisor behavior [47], meaning their emotional and psychological perceptions are less influenced by supervisors. As a result, compared to postgraduate students with high power distance orientation, the positive effect of supervisors’ humble leadership on basic psychological need satisfaction is relatively weaker. Related research confirms that individuals with low power distance orientation are less affected by humble leadership in terms of basic psychological need satisfaction compared to those with high power distance orientation [45].

**Hypothesis** **3** **(H3).** *Power distance orientation positively moderates the relationship between supervisors’ humble leadership and basic psychological need satisfaction, with the effect being stronger for individuals with higher power distance orientation than for those with lower orientation*.

### 2.4. The Moderated Mediation Effect

Further, based on hypotheses H2 and H3, we propose a moderated mediation model, wherein basic psychological need satisfaction mediates the impact of supervisors’ humble leadership on postgraduate students’ well-being, but the extent of this impact is moderated by the power distance orientation of the postgraduates. Postgraduates with a high power distance orientation tend to respond more sensitively to supervisors’ humble leadership, thus more fully satisfying their basic psychological needs and enhancing their well-being. Conversely, postgraduates with a low power distance orientation exhibit a less sensitive response to supervisors’ humble leadership, somewhat diminishing the positive effect on basic psychological need satisfaction and subsequently constraining substantial enhancements in well-being. Previous research also indicates that power distance orientation influences subordinates’ interpretations of and reactions to superior behavior, thereby moderating the mediating role of basic psychological need satisfaction in the relationship between leadership and individual attitudes [48]. Based on this, this study proposes a hypothesis of moderated mediation:

**Hypothesis** **4** **(H4).** *Power distance orientation moderates the indirect effect of supervisors’ humble leadership on postgraduate students’ well-being via basic psychological need satisfaction, with the effect being stronger for students with higher power distance orientation*.

**Figure 1:**
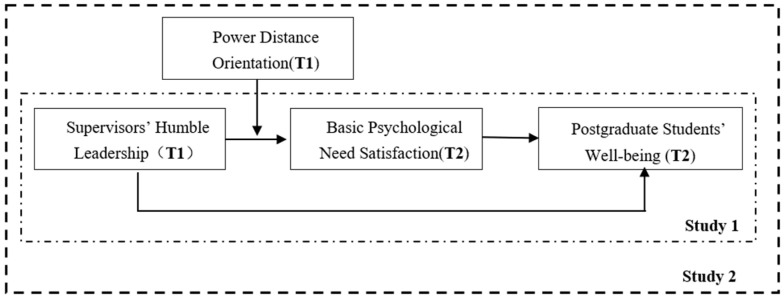
Conceptual model.

To sum up, the theoretical model of this study is shown in Figure 1. This paper aims to investigate the relationship and underlying mechanisms between supervisors’ humble leadership and postgraduate students’ well-being. Study 1, using a survey experiment, explores the immediate effect of supervisors’ humble leadership on postgraduate students’ well-being through basic psychological need satisfaction, testing H1 and H2. Study 2, using a multi-timepoint survey, validates the delayed effect of supervisors’ humble leadership on postgraduate students’ well-being through basic psychological need satisfaction and examines the moderating role of power distance orientation in the indirect impact of supervisors’ humble leadership on postgraduate students’ well-being through basic psychological need satisfaction, namely H3 and H4.

## 3. Study 1: Survey Experiment

### 3.1. Method 

#### 3.1.1. Participants and Design

This study employed a single-factor between-subjects design and used G*Power 3.1 software [49] to calculate that, at α = 0.05 and power = 0.8, the required sample size to achieve a medium effect size was 128. The study recruited 150 university students through Creado (www.creado.com, accessed on 1 March 2024). After excluding three participants who failed the attention check, 147 participants were included in the data analysis. The study sample comprises 61 males and 86 females. In terms of disciplinary backgrounds, 59 participants are from the fields of economics, management, and law, 9 from literature, history, and philosophy, 25 from education and arts, and 54 from engineering, science, and medicine. Participants were randomly assigned to either the experimental group (n = 72, females = 44) or the control group (n = 75, females = 43), read the experimental materials and assumed the roles described therein, and then completed manipulation check questions, scales measuring basic psychological need satisfaction and postgraduate students’ well-being scales, and other related control variables.

#### 3.1.2. Measures

Manipulation of supervisors’ humble leadership: Drawing from the study by Liborius and Kiewitz (2022) [50], materials for manipulating supervisors’ humble leadership were developed. High (low) supervisors’ humble leadership was described as follows: “Imagine you are a postgraduate student at R University. In your daily interactions, you notice that your supervisor often (never) seeks your or others’ opinions on key issues. Your supervisor admits (does not admit) when he/she does not know how to handle certain matters and acknowledges (does not acknowledge) that others may have more knowledge and skills. Furthermore, your supervisor notices (never notices) your strengths and contributions, frequently (never) praises you, and is willing (refuses) to accept your new ideas and suggestions. Facing such a supervisor, I would feel...?”

Basic psychological need satisfaction: This study utilized a 9-item three-dimensional scale developed by Sheldon (2001) [51] to measure basic psychological need satisfaction. Responses were recorded on a 5-point Likert scale ranging from 1 (strongly disagree) to 5 (strongly agree). In this study, the scale had a Cronbach’s α of 0.80.

Postgraduate students’ well-being: Adapted from the overall well-being questionnaire developed by Zheng (2015) [52], and customized for the context of postgraduate students. Example items included: “Overall, I am satisfied with the research tasks I am undertaking” and “In general, I feel affirmed and confident in myself”. Responses were collected using a 5-point Likert scale, ranging from 1 (strongly disagree) to 5 (strongly agree). In this study, the scale of Cronbach’s α was 0.89.

Manipulation checks for supervisors’ humble leadership (high vs. low): Based on the study by Liborius and Kiewitz (2022) [50], participants were asked three questions: “The supervisor admits when students have more knowledge and skills”, “The supervisor appreciates students’ contributions”, and “The supervisor accepts students’ suggestions”. Responses were recorded on a 5-point Likert scale from 1 (strongly disagree) to 5 (strongly agree).

### 3.2. Results

#### 3.2.1. Manipulation Check

This study employed an independent-sample t-test to verify the successful manipulation of supervisors’ humble leadership. The results indicate that, under the high-level supervisors’ humble leadership scenario, participants’ perceptions of supervisors’ humble leadership (*M* = 3.717, *SD* = 0.453) are significantly higher than in the low-level scenario (*M* = 1.680, *SD* = 0.479), *t* (145) = 26.464, *p* < 0.01, Cohen’s d = 4.370, confirming the effective manipulation of the experiment.

#### 3.2.2. Hypotheses Testing

**Figure 2:**
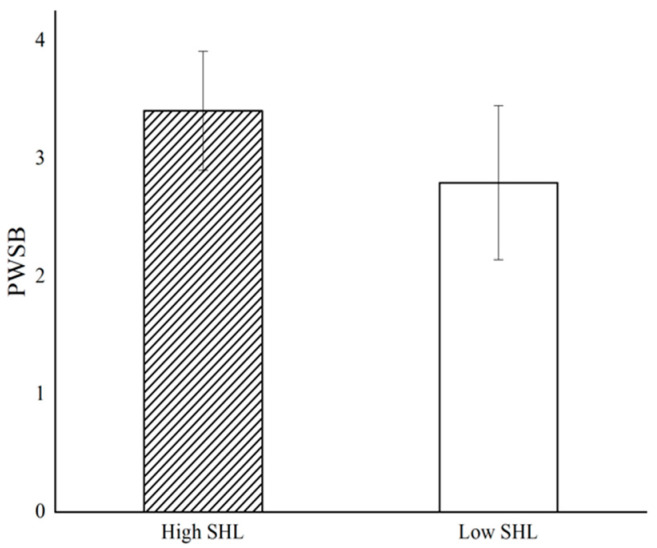
Impact of supervisors’ humble leadership (SHL) on postgraduate students’ well-being (PSWB).

**Figure 3:**
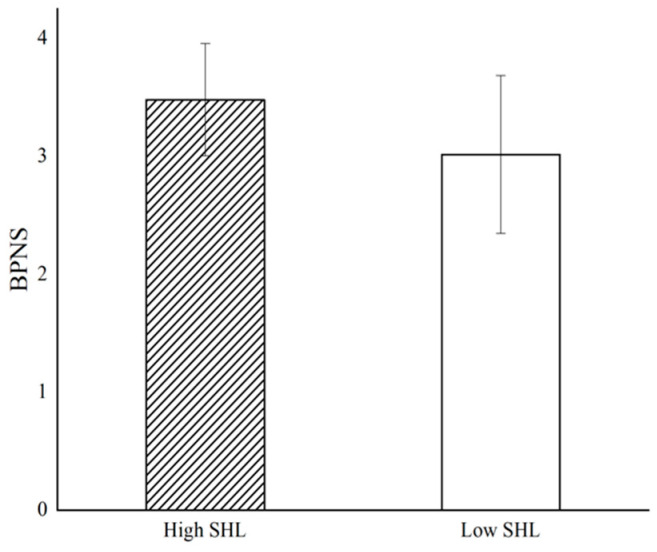
Impact of supervisors’ humble leadership (SHL) on basic psychological need satisfaction (BPNS).

According to the results of the independent-sample *t*-test (see Figure 2), postgraduate students’ well-being under high supervisors’ humble leadership scenarios (*M* = 3.407, *SD* = 0.505) is significantly higher than in low supervisors’ humble leadership scenarios (*M* = 2.796, *SD* = 0.653), *t* (145) = 6.333, *p* < 0.001, Cohen’s d = 1.047), supporting H1. Similar to the results for postgraduate students’ well-being (see Figure 3), basic psychological need satisfaction under high supervisors’ humble leadership scenarios (*M* = 3.480, *SD* = 0.476) is significantly different from low scenarios (*M* = 3.016, *SD* = 0.670), *t* (134) = 4.855, *p* < 0.001, Cohen’s d = 0.798.

To verify the mediating role of basic psychological need satisfaction, we used the SPSS PROCESS (Model 4) with bootstrap for 5000 resamples to estimate the 95% confidence interval. The results indicate that basic psychological need satisfaction positively predicted postgraduate students’ well-being after controlling for gender and age, and the indirect effect of supervisors’ humble leadership on postgraduate students’ well-being through basic psychological need satisfaction is 0.179, *SE* = 0.033, 95% CI [0.114, 0.246], thus confirming H2.

### 3.3. Discussion

Study 1 utilized a survey experiment to examine the positive immediate impact of supervisors’ humble leadership on postgraduate students’ well-being and preliminarily tested the mediating effect of basic psychological need satisfaction, thus validating H1 and H2. However, as the data in Study 1 were collected under experimental conditions, there may be discrepancies with the complexity of real-world scenarios. To enhance the external validity of the findings, expanding the sample size was necessary. Additionally, the single-timepoint experimental design limits the ability to capture the dynamic interactions between variables, warranting the use of a multi-timepoint survey to increase the robustness of the results. Furthermore, randomly assigning participants to low or high power distance orientation scenarios without accounting for individual differences in power distance orientation may obscure the moderating role of this variable. As a result, Study 1 did not empirically test the moderating effect of power distance orientation. Therefore, in Study 2, a multi-timepoint survey was employed in real-world settings to validate the conclusions of Study 1 and examine the moderating effects of power distance orientation (H3, H4). This will provide cross-validation for the findings derived from the experimental study.

## 4. Study 2: Survey Questionnaires 

### 4.1. Method

#### 4.1.1. Participants and Procedures

To test the hypothesis model, data were collected using a convenient sampling method. Specifically, researchers recruited 100 Chinese postgraduate students from several universities to participate in this study, with each student then randomly inviting 4 to 6 classmates to join (thus, both the postgraduates and their invited classmates participated as subjects in the survey). The survey for this study was designed on the Questionnaire Star (wjx.cn) and distributed via a WeChat link; each survey was initially sent by researchers to 100 postgraduates via WeChat, who then forwarded it to their classmates. This study utilized a data collection method over two timepoints, with an interval of one month between each. At Time 1 (T1), postgraduate students filled out demographic information and evaluated their perception of supervisors’ humble leadership as well as their own power distance orientation. A total of 470 questionnaires were received. At Time 2 (T2), postgraduate students assessed their basic psychological need satisfaction and postgraduate student well-being over the past month. The questionnaires from both times were combined, resulting in 431 valid responses. In the final valid sample, approximately 43.6% of the respondents were male, and about 95.4% of the postgraduate students were between the ages of 22 and 30. Postgraduate students in the fields of economics, management, and law accounted for 35.7%. Regarding the academic titles of their supervisors, approximately 56.4% were professors and 34.3% were associate professors. About 32.9% of the postgraduate students were studying at 985 universities.

#### 4.1.2. Measures

This study adopted well-established scales from both domestic and international sources. The survey was conducted in Chinese. To ensure consistency with the original English versions, a rigorous translation-back translation procedure was followed. To enhance the specificity and appropriateness of the questionnaire items, three postgraduate students were invited to participate in a pilot survey before the formal study, and the questionnaire was revised based on their feedback. Apart from statistical variables, all scales used a 5-point Likert scale ranging from 1 = strongly disagree to 5 = strongly agree.

Supervisors’ humble leadership: This study used the 9-item three-dimensional Humble Leadership Scale developed by Owens (2013) [14], which was contextually adapted for the postgraduate student population. Example items included “My supervisor acknowledges that students have more knowledge and skills than they do” and “My supervisor frequently praises students’ strengths”. The scale’s Cronbach’s α was 0.885, indicating good reliability.

Power distance orientation: The measure for power distance orientation used a unidimensional 6-item questionnaire developed by Dorfman and Howell (1988) [43]. In this study, the scale of Cronbach’s α was 0.896.

Postgraduate students’ well-being: This study also utilized the well-being scale developed by Zheng (2015) [52]. In this study, Cronbach’s α was 0.919.

Basic psychological need satisfaction: This study also used the scale developed by Sheldon (2001) [51]. In this study, Cronbach’s α was 0.873. The relevant content can be found in Table 1.

Control variables: Research has indicated that the effectiveness of a supervisor’s leadership was related to the supervisor’s career stage, discipline differences, and gender differences among postgraduates [53]. Accordingly, this paper included variables such as student gender, discipline (economics, law, humanities, philosophy, science, engineering, medical sciences), supervisory titles (professor, associate professor, lecturer), and university categories (985 universities (the ‘985 Project’, launched in 1998, aimed to create world-class research universities in China through government funding and support. It includes 39 top institutions, like Tsinghua and Peking University), and 211 (non-985) universities (the ‘211 Project’, launched in 1995, aimed to strengthen 100 key universities and disciplines. It includes 112 institutions, with a broader range of key universities across China)) as control variables.

#### 4.1.3. Data Analysis

All statistical analyses in this study were conducted using SPSS 26.0 software. First, SPSS 26.0 was used to conduct tests for common method bias, descriptive statistical analysis, correlation analysis, and reliability analysis. Subsequently, hierarchical regression analysis and PROCESS (Model 7) in SPSS 26.0 were employed to test the hypotheses.

### 4.2. Results

#### 4.2.1. Common Method Variance Check

To avoid common method bias, we conducted Harman’s single-factor test. The results indicate that the largest factor accounts for 27.983% of the variance, which is below the threshold of 40% [54], suggesting that there is no significant common method bias in this study.

#### 4.2.2. Descriptive Statistics and Effect Sizes

Means, SDs, and correlations among variables are presented in Table 2. Specifically, supervisors’ humble leadership at T1 is significantly positively correlated with basic psychological need satisfaction at T2 (*r* = 0.532, *p* < 0.01) and postgraduate students’ well-being at T2 (*r* = 0.457, *p* < 0.01); basic psychological need satisfaction at T2 is also significantly positively correlated with postgraduate students’ well-being at T2 (*r* = 0.552, *p* < 0.01). These results provide preliminary support for the hypotheses.

#### 4.2.3. Hypothesis Testing

##### Direct Effect Testing

Table 3 presents the hierarchical regression results for the main study variables. As shown in Model 6, the coefficient for the effect of supervisors’ humble leadership at T1 on postgraduate students’ well-being at T2 is significantly positive (β = 0.450, *p* < 0.001). This finding indicates that SHL has a positive direct effect on postgraduate students’ well-being, thereby supporting H1.

##### Mediating Effect Testing

To examine the mediating role of basic psychological need satisfaction at T2 in the relationship between supervisors’ humble leadership at T1 and postgraduate students’ well-being at T2, this study followed Preacher and Hayes’ procedure for mediation testing. Results from Model 2 in Table 3 show a significant positive correlation between supervisors’ humble leadership at T1 and basic psychological need satisfaction at T2 (β = 0.506, *p* < 0.001).

When adding basic psychological need satisfaction at T2 to the regression model, results from Model 7 in Table 3 show that basic psychological need satisfaction at T2 positively predicts postgraduate students’ well-being at T2 (β  = 0.433, *p* < 0.01), and the positive impact of supervisors’ humble leadership at T1 on postgraduate students’ well-being at T2 remains significant (β  = 0.231, *p* < 0.01). Consequently, basic psychological need satisfaction at T2 mediates the relationship between supervisors’ humble leadership at T1 and postgraduate students’ well-being at T2 among postgraduates, supporting H2.

##### Moderating Effect Testing

To test whether power distance orientation at T1 moderates the relationship between supervisors’ humble leadership at T1 and basic psychological need satisfaction at T2, a moderating effect analysis was conducted. Results from Model 3 in Table 3 show that the interaction between supervisors’ humble leadership at T1 and power distance orientation at T1 significantly predicts basic psychological need satisfaction at T2 (β = 0.160, *p* < 0.001). This suggests that power distance orientation at T1 positively moderates the effect of supervisors’ humble leadership on basic psychological need satisfaction at T2.

**Figure 4:**
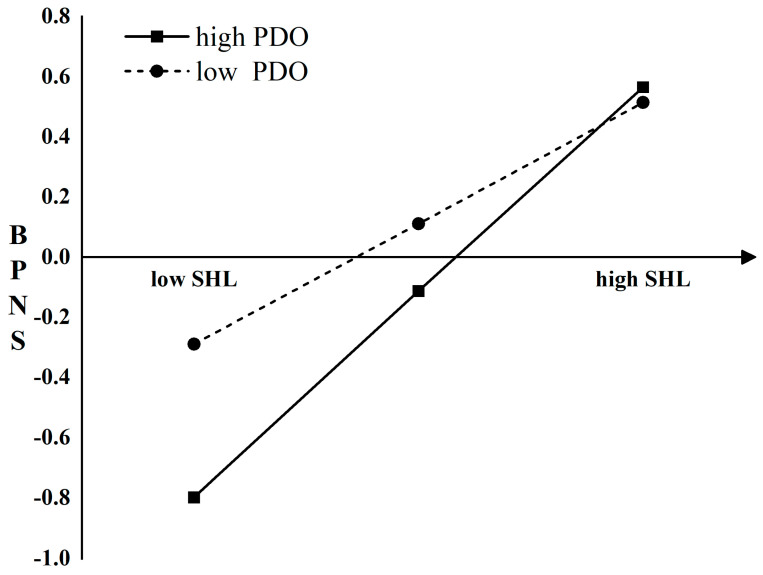
Power distance orientation (PSWB) moderates the relationship between supervisors’ humble leadership (SHL) and basic psychological need satisfaction (BPNS).

By adjusting the mean of power distance orientation at T1 by ±1 standard deviation, we divided the data into two groups: high and low power distance orientation. The results in Figure 4 indicate that the positive effect of supervisors’ humble leadership on basic psychological need satisfaction is stronger for students with high power distance orientation at T1 compared to those with low power distance orientation, supporting H3. 

##### Moderated Mediation Effect Testing

To examine the full theoretical model, Hayes’ (2013) method was used with Model 7 from the PROCESS plugin in SPSS. The results indicate that supervisors’ humble leadership at T1 positively predicts basic psychological need satisfaction at T2 (β = 0.509, SE = 0.042, 95% CI [0.456, 0.621]). Furthermore, the interaction between supervisors’ humble leadership and power distance orientation at T1 significantly predicts basic psychological need satisfaction at T2 (β = 0.138, SE = 0.037, 95% CI [0.065, 0.211]). 

These results suggest that the effect of supervisors’ humble leadership on postgraduate students’ well-being through basic psychological need satisfaction is moderated by power distance orientation. Table 4 shows that a simple effect analysis revealed that the mediating effect of basic psychological need satisfaction is stronger at high power distance orientation (β = 0.293, CI [0.194, 0.394]) and weaker at low power distance orientation (β = 0.174, CI [0.112, 0.245]), supporting H4 and the moderated mediation model.

## 5. General Discussion

Finding effective strategies to boost postgraduate well-being, cultivate resilience, and foster a positive outlook in facing challenges is increasingly emphasized by both practitioners and scholars. This study, grounded in positive psychology and SDT, investigates how supervisors’ humble leadership can activate basic psychological need satisfaction and enhance postgraduate students’ well-being. Additionally, analyzing the moderating role of power distance orientation reveals that the effectiveness of supervisors’ humble leadership on basic psychological need satisfaction depends significantly on power distance orientation—a critical boundary condition. Study 1 (survey experiment) validated the immediate impact of supervisors’ humble leadership on basic psychological need satisfaction and well-being. Study 2 (survey questionnaires) validated the delayed effect of supervisors’ humble leadership on basic psychological need satisfaction and well-being, and further tested the mediating role of basic psychological need satisfaction in the relationship between supervisors’ humble leadership and postgraduate students’ well-being, moderated by power distance orientation, providing further support for the theoretical model of this research.

### 5.1. Theoretical Implications

First, this study explores the immediate and delayed impact of supervisors’ humble leadership on postgraduate students’ well-being, expanding the research on antecedents of postgraduate students’ well-being. Existing studies on postgraduate students’ well-being have largely focused on factors such as learning interventions [55,56] and social support [8,11], with limited exploration of supervisors’ humble leadership. The two studies in this research both demonstrate a significant positive relationship between supervisors’ humble leadership and postgraduate students’ well-being. According to general psychological principles, individuals who maintain a more realistic self-view tend to have better mental health and higher overall well-being [14]. Humility involves having a grounded view of oneself and others, as well as an open mindset for learning [21], which can be observed by others in interpersonal interactions [14]. The interpersonal nature of the supervisor-student relationship enables students to develop an openness to new paradigms and a correct self-awareness under the influence of humble leadership. Self-acceptance and openness are particularly important for postgraduate students’ well-being [55]. Another well-being dimension related to humble leadership is the individual’s experience of positive emotions. Humble supervisors are often skilled at recognizing and appreciating the strengths and achievements of others [14], enhancing subordinates’ positive subjective emotional experiences [57]. Under the influence of this leadership, students are more likely to experience positive emotions, thereby improving their well-being.

Secondly, this study is based on SDT and explores the mediating role of basic psychological need satisfaction, clarifying the internal mechanisms by which supervisors’ humble leadership influences postgraduate students’ well-being, and expanding the theoretical perspective of humble leadership research. Previous research on the mechanisms of humble leadership primarily analyzed its impact on subordinate attitudes and behaviors through individual-level psychological contracts [58], psychological safety [59], and subordinate conflicts [60] from the perspectives of social exchange theory, social information processing theory, and resource-based theory, overlooking the role of basic psychological need satisfaction. Consequently, based on SDT, we explored how supervisors, as key environmental factors for postgraduate students, create an autonomy-supportive environment through their humble leadership, thereby addressing this theoretical gap. The results showed that the supervisors’ humble leadership, by affecting basic psychological need satisfaction, significantly enhanced postgraduate students’ well-being, with both immediate improvements and lasting positive effects. This conclusion not only provides new theoretical insights for research on humble leadership, but also further elucidates the impact of supervisors’ humble leadership on postgraduate students’ well-being, identifying its mediating effects.

Finally, the moderating effect of power distance orientation was examined from a cultural values perspective, providing new insights into the relationship between supervisor leadership and students’ psychological states. Study 2 confirmed that power distance orientation moderates the positive relationship between supervisors’ humble leadership and basic psychological need satisfaction in postgraduates, aligning with Deci and Ryan’s (2001) cross-cultural findings on SDT, which highlight the cultural variance in the impact of leadership autonomy support on basic psychological need satisfaction [19]. Our study further explored this moderating effect in a Chinese postgraduate sample, adding nuance to SDT’s cross-cultural application. Specifically, the results show that supervisors’ humble leadership has a stronger positive impact on basic psychological need satisfaction for students with high power distance orientation compared to those with low power distance orientation. This may be due to the traditional Chinese value of “respecting teachers”, which emphasizes respect and obedience to authority figures. This cultural norm shapes individuals’ high power distance orientation, making them more inclined to accept unequal teacher-student relationships. When supervisors display humble leadership, it challenges these students’ expectations, evoking a sense of unexpected respect and understanding that deeply fulfills their basic psychological need satisfaction. In contrast, low power distance orientation students expect a more egalitarian relationship with their supervisors. While supervisors’ humble leadership can also meet their basic psychological need satisfaction, the alignment of this behavior with their existing expectations of equality and mutual respect means that their response is less pronounced compared to high power distance orientation students. This difference suggests that students with high power distance orientation may benefit more from supervisors’ humble leadership, as they are more sensitive to changes in power dynamics.

### 5.2. Practical Implications

Practices such as Frankl’s “logotherapy” [61] have proven highly effective in helping individuals facing severe challenges, distress, anxiety, or even suicidal tendencies to rediscover the meaning of life. Based on this, the findings of this study provide significant guidance on how to enhance postgraduate students’ well-being and strengthen their ability to face challenges. The following sections provide targeted recommendations for various stakeholders based on this study’s results.

#### 5.2.1. Implications for Postgraduate Supervisors

First, supervisors should recognize their dual role as both academic mentors—guiding postgraduates in developing scientific spirit and innovation skills—and as life mentors, setting an example to help students establish a correct worldview and values. Second, supervisors should practice humble leadership, set aside their authoritative demeanor, focus on students’ mental and physical health, actively engage with students, and guide them with appreciation, encouragement, and sincerity to enhance their well-being and foster a positive outlook. Lastly, this study shows that supervisors’ humble leadership has varying positive effects on individuals with different power distance orientations, with the impact being more pronounced for those with a higher power distance orientation. Therefore, in the context of cultures that emphasize respect for teachers, supervisors should adopt a humble leadership style to more effectively enhance students’ basic psychological need satisfaction and overall well-being.

#### 5.2.2. Implications for Postgraduates

First, postgraduates should maintain a proactive approach to their studies and life, develop healthy habits, and pursue hobbies outside academics. Practicing stress management techniques like meditation and deep breathing can also enhance well-being. Second, postgraduates should assertively reject inappropriate behavior from supervisors and, if needed, use formal channels within their department to safeguard their rights.

#### 5.2.3. Implications for Universities and Educational Authorities

Universities and educational authorities should also strengthen the selection and management of postgraduate supervisors, establish regular grievance mechanisms, and encourage postgraduates to proactively report any misconduct by supervisors to their departments or universities. Additionally, the results indicate that satisfying basic psychological need satisfaction effectively enhances postgraduate students’ well-being. Educators should thus meet the autonomy, competence, and relatedness needs of postgraduates, create environments that support basic psychological need satisfaction, and optimize the postgraduate educational experience.

### 5.3. Limitations and Future Directions

This study used survey experiments and multi-timepoint surveys to elucidate the internal mechanisms and boundary conditions under which supervisors’ humble leadership affects postgraduate students’ well-being; yet, it has certain limitations. Firstly, data collection was solely based on self-reports from postgraduates, which may introduce some biases due to subjectivity and memory. To overcome these limitations, future studies could include supervisors as research subjects and collect data through supervisor-postgraduate matching. Second, due to the focus of the research questions, this study examines the satisfaction of basic psychological needs as a whole. However, other studies suggest that exploring the uniqueness of each psychological need from a unidimensional perspective is essential [62]. Future studies can further investigate how supervisors’ humble leadership enhances graduate students’ well-being from the perspectives of autonomy, competence, and relatedness needs. Moreover, all variables in this study are at the individual level, and no multi-level analysis has been conducted. In real-world scenarios, nested data may exist, where a single supervisor supervises multiple graduate students, and supervisors may engage in group-level supervisory behaviors when overseeing several students simultaneously. Based on this, future research could employ multi-level analysis, selecting team-level variables to explore in depth the mechanisms through which supervisors’ humble leadership affects postgraduate students’ well-being at both organizational and individual levels.

## Figures and Tables

**Table 1:** Overview of scales and measures used in the study.

Measure	Scale Developer	Example Items	**Cronbach′s** α
SHL	Owens (2013)	My supervisor frequently praises students’ strengths	0.885
PDO	Dorfman Howell (1988)	When assigning learning tasks, the supervisor does not need to consult the students’ opinions	0.896
PSWB	Zheng (2015)	I am satisfied with my work responsibilities	0.919
BPNS	Sheldon (2001)	My choices were based on my true interests and values	0.873

Note: SHL = supervisors’ humble leadership; BPNS = basic psychological need satisfaction; PSWB = postgraduate students’ well-being; PDO = power distance orientation.

**Table 2:** Means, SDs, and correlations among variables.

Variable	1	2	3	4	5	6	7	8
1. Gender (T1)	—							
2. Discipline (T1)	−0.243 **	—						
3. Supervisory titles (T1)	−0.075	0.007	—					
4. Universities categories (T1)	−0.093 *	0.292 **	0.053	—				
5. SHL (T1)	0.105 *	−0.155 **	0.121 *	−0.128 **	—			
6. BPNS (T2)	0.100 *	−0.183 **	0.059	−0.179 **	0.532 **	—		
7. PSWB (T2)	0.054	−0.054	0.055	−0.143 **	0.457 **	0.552 **	—	
8. PDO (T1)	−0.222 **	0.238 **	0.184 **	0.061	0.009	−0.079	0.054	
M	1.560	2.240	1.560	2.00	3.580	3.710	3.654	2.789
SD	0.496	1.138	0.744	0.816	0.741	0.647	0.619	0.904

Note: Gender: 1 = males, 2 = females. Discipline: 1 = economics, management, and law; 2 = humanities and philosophy; 3 = science and engineering; 4 = medicine and life sciences. Supervisory titles: 1 = professor; 2 = associate professor; 3 = lecturer; 4 = other. University categories: 1 = 985 universities; 2 = 211 universities (non-985); 3 = other universities. SHL = supervisors’ humble leadership; BPNS = basic psychological need satisfaction; PSWB = postgraduate students’ well-being; PDO= power distance orientation. * *p* < 0.05, ** *p* < 0.01.

**Table 3:** Results of hierarchical regression analyses.

Variable	BPNS (T2)			PSWB (T2)			
	Model 1	Model 2	Model 3	Model 4	Model 5	Model 6	Model 7
Gender (T1)	0.061	0.021	0.005	0.045	0.011	0.009	0.000
Discipline (T1)	−0.128 *	−0.073	−0.047	−0.002	0.069	0.047	0.079
Supervisory titles (T1)	0.071	0.004	0.017	0.066	0.026	0.006	0.004
Universities categories (T1)	−0.140 **	−0.092 *	−0.07	−0.142 **	−0.064	−0.099 *	−0.059
SHL (T1)		0.506 ***	0.538 ***			0.450 ***	0.231 ***
BPNS(T2)					0.551 ***		0.433 ***
PDO (T1)			−0.116 **				
SHL*PDO (T1)			0.160 **				
*R^2^*	0.059	0.296	0.301 ***	0.026	0.312	0.218	0.349
*∆* *R^2^*	0.050	0.293	0.315	0.017	0.304	0.209	0.340
*F*	6.653 ***	147.231 ***	8.016 ***	2.881	176.142 ***	104.306 ***	85.362 ***

Note: SHL = supervisors’ humble leadership; BPNS = basic psychological need satisfaction; PSWB = postgraduate students’ well-being; PDO= power distance orientation. * *p* < 0.05, ** *p* < 0.01, *** *p* < 0.001.

**Table 4:** Results of bootstrap analyses.

Conditional Indirect Effect	Effect	SE	95% CI
M + 1SD	0.293	0.051	[0.194, 0.394]
M	0.233	0.037	[0.161, 0.309]
M − 1SD	0.174	0.034	[0.112, 0.245]
**Index of moderated mediation**			
PDO	0.060	0.022	[0.019, 0.104]

Note: N = 431; bootstrap sample size = 5000; CI = confidence interval; PDO = power distance orientation.

## Data Availability

The datasets analyzed during the current study are available from the corresponding author on reasonable request.
